# Book review: Teaching language online: A guide for designing, developing, and delivering online, blended, and flipped language courses

**DOI:** 10.3389/fpsyg.2022.1011021

**Published:** 2022-11-15

**Authors:** Lina Chi, Fang Wang

**Affiliations:** ^1^School of Foreign Studies, Yangtze University, Jingzhou, China; ^2^School of Cultures, Languages, and Linguistics, The University of Auckland, Auckland, New Zealand; ^3^School of Foreign Languages, Jingzhou University, Jingzhou, China

**Keywords:** online language courses, blended language courses, flipped language courses, online teaching, design guidelines

As information technology has been widely used in language education over the past decades, online language learning is gradually being accepted as an effective way to provide learners with a more interactive and convenient language learning environment (Lee, 2016; Kohnke and Moorhouse, 2022). Especially during the spread of COVID-19 globally, online teaching on digital platforms has been extensively employed as a substitute for face-to-face classroom instruction. In the book *Teaching Language Online: A Guide for Designing, Developing, and Delivering Online, Blended, and Flipped Language Courses*, Victoria Russell and Kathryn Murphy-Judy provides a well-developed and timely guide for online language teaching and learning (Russell and Murphy-Judy, 2020). Based on the authors' years of non-traditional teaching experience, they aim to offer language teachers, professors, and K-12 language educators around the world a guide conducive to integrating instructional technology in online practical teaching and improving teacher professional development. It is intended to ultimately allow language teaching practitioners to create an effective, productive, and successful online teaching environment.

Besides the Introduction and Conclusion chapters, the book consists of five chapters that separately end with “Key Takeaways,” “Discussion Questions,” and “Suggestions for Further Reading” sections to provide online instructors with further thinking and exploration. This organizing style integrates all the chapters as a concert as well as grants each chapter a solo. Therefore, readers can capture relevant resources in a short time.

The two initial chapters cover the basics of analyzing, designing, and developing online courses. Chapter 1 explains the instructional design process, and accentuates the necessity of conducting needs analysis before carrying out online teaching. ADDIE, the model used throughout the whole book, proposed by Gustafson and Branch (2002), is a time-tested process model, representing analysis, design, delivery, implementation, and evaluation to guide online language teaching and learning. It offers a solid foundation for creating online learning programs, courses, and modules. The authors focus on the analysis and design phases of ADDIE by exploring further details and with specific regard to the field of online language education. Adopting an inquiry-based format, the analysis section in the chapter introduces the most important considerations that need to be investigated before building a successful design, which helps designers capture the specific demand of online learners. Then the “backward design” model is employed to pay special attention to what learners actually gain from the instruction and what they can do after that, rather than how to cover all the content during the allocated instructional period. Consequently, the analysis and design procedure guarantees the learners' required learning content. The theories, research, and models in instructional design, distance and online instructional design, and computer-assisted language learning (CALL) presented in this chapter are culled from the effective practices of veteran online language educators. The authors mainly address secondary and postsecondary educational settings and adult and young adult learners, but they believe that corporate, military, and government educators may also benefit from this content.

Chapter 2 continues with the second “D” of ADDIE, which contains the development of the learner, the learning management system (LMS), the course structure, and interaction and course activities, focusing on the strategies, tools, technologies, and procedures for developing successful online language learners. Learners as the core should be considered as the primary factor of online course development. Unquestionably, learners expect to have the same high-quality learning experiences and achieve successful learning effects in online learning as in traditional learning. Indeed, the development of online language learners' competence and attitude is not developed by themselves alone but also reinforced by the guidance of competent online language instructors. Thus, the authors make it clear that instructional designers should prioritize students' development as language learners and online learners, especially toward their self-regulation and autonomy. In this perspective, the three types of interactions (learner–instructor, learner–learner, and learner–content) are claimed to be helpful for creating an interactive online learning environment. This part is valuable as it guides the instructors and learners to interact in a more efficient way through online course humanization.

Chapter 3 is about implementation, which is the key to effective online teaching, aiming to guide language educators to develop their key knowledge of online teaching pedagogy to ensure the successful delivery of their online language teaching. It starts by discussing the competencies to support online language pedagogy, which is the foundation of all online language educators. Then, listing 10 guidelines (e.g., focusing on meaning, facilitating classroom interaction, avoiding mechanical activities) with clear examples, the authors show us how communicative language teaching (CLT) and the core practices (including teachers' instructional skills, actions, behaviors, and techniques) that help to advance students' language learning can be implemented in online, blended, or flipped courses. Incorporating all the 10 guidelines in designing, developing, and delivering online courses requires some forethought and implementation of instructional technologies, but employing them appropriately will facilitate learners' communication and promote their language acquisition process. The CLT approach, which ensures online, blended, and flipped language courses are taught communicatively, is prominently emphasized in the guidelines. For instance, Guideline 7 “engage students in open-ended communication where they can create with language” (p. 143), which ensures that students communicate freely and creatively in three modes of communication: interpretive, interpersonal, and presentational. The chapter also introduces how to apply good teaching practices to online teaching and training to provide a guiding learning environment. The top priority for teachers is to understand the teaching practices that help promote students' language acquisition, enhance students' engagement, and develop the awareness of how to implement these practices in online teaching. After all, language courses require teachers to impart cultural knowledge through literature, history, and geography, and to teach language in a meaningful environment.

Chapter 4 sketches out an overview of teacher professional development opportunities and provides open educational resources for online language teachers, emphasizing the importance of seeking professional development by promoting instructors' skills and creating their toolbox for teaching online. Lacking a benchmark for online teaching is a non-negligible issue for the instructors' transition to online teaching (Tschida et al., 2016), so the authors share information on several language resource centers that provide open-access professional development materials for language teachers. For example, they not only point out resources available from different organizations for content development, curriculum selection, and online language teaching approaches, but also recommend specific professional organizations that concentrate on CALL, remote language teaching and learning, and online teaching and learning culture. Although many materials, resources, activities, and professional development opportunities are offered in this chapter, the authors advise the teachers to select the most suitable ones according to their actual situation such as their teaching content, teaching skill level, the students, and the online teaching condition.

In Chapter 5, to help language instructors make most of the findings of research to their online courses, Russell and Murphy-Judy present research on online language teaching and its pedagogical implications. They select the studies with themes on student and teacher satisfaction, class size, language anxiety, best practices, social presence, learner connectedness, and assessment, all of which are topics that are fundamental to online course delivery. The research is presented in a clear and jargon-free manner to allow readers to easily understand what works and why in online, blended, and flipped language environments. Each study reviewed is followed by pedagogical significance and practical instances for improving online practices, which can help online language instructors create an environment that affords more meaningful, effective, and enjoyable learning experiences for language learners. Effective and reliable assessment results are key to examining whether the educational goals have been achieved or not. Remote assessment is a challenge for the instructors, the learners, and even the technical infrastructure, so this chapter ends by introducing research-based assessment techniques. The chapter ends by introducing research-based assessment techniques. Actually, in the ADDIE model, the evaluation phase is the beginning of new and better practices based on evaluation data and an examination of the research. The concluding chapter is not an end, but rather a new beginning. It opens the door to language education and shows us the bright future of online teaching. Online language education is a new norm not merely because of the COVID-19 pandemic, but also because of its advantages of flexibility, information accessibility, global reach, equity, innovation, and efficiency (Xie et al., 2020), with the fact that enrollment in online courses and programs keeps growing globally. As the authors rightly point out, advanced technologies (e.g., data analytics, adaptive learning software, etc.) will serve as a facilitator for students' language learning if they are properly integrated into the instructional systems. But the authors also caution that successful online language teaching requires instructors to tailor their courses for each student. Furthermore, language educators who transition from traditional to online instruction should pay much attention to their professional development by acquiring knowledge of online language pedagogy and instructional technologies. The authors claim that armed with the knowledge, skills, and practices of effective online language teaching, the instructors will be expected to design and deliver successful online courses.

This book takes readers to enjoy a journey into online language teaching and learning. It is of great value as a guide for online language teaching. Being good language teachers in the traditional face-to-face environment does not mean that it will naturally be the same in an online context. The authors, Victoria Russell and Kathryn Murphy Judy, aim at helping language instructors successfully transition from traditional to online instruction in a more efficient way. Overall, the book is of both practical and theoretical significance. Practically, as mentioned above, the authors have provided abundant eResources and many practical tips and suggestions throughout the book for online course development and delivery as well. In addition, in a down-to-earth manner, the authors also provide illuminating insights (e.g., communicating with other online language instructors through professional development activities such as conferences, seminars, or workshops online or offline to defeat their feelings of helplessness or isolation) for language teachers into how they can achieve long-term professional development to ensure their success in online instruction. Meanwhile, they offer clear suggestions as to such questions as who can help teachers' professional development, and what resources could be used to effectively promote teachers' professional development.

Given the consideration of professional development in a technology-mediated language context, we believe the Technological Pedagogical Content Knowledge (TPACK) framework, extended by Mishra and Koehler (2006) on the basis of Shulman's (1986) idea of pedagogical content knowledge (PCK), will be a useful guide for integrating technology in teachers' practices. It is generally challenging for teachers to apply technology successfully in the language teaching and learning process. Integrating the three interrelated elements (i.e., technology, pedagogy, and content knowledge) is essential but most teachers still lack an in-depth understanding of collaborative curriculum design even though they have ample knowledge of each element (Boschman et al., 2014).

Though not necessarily designed as a research-oriented reference book, it provides implications for theory building and future research directions. For example, in Chapter 5, the authors provide a systematic review of online language teaching, which not only renders abundant pedagogical implications but also shows that online language instruction is a social and complex phenomenon mediated by teacher and learner affect, which, in turn, is mediated by a large set of contextual and individual factors. Therefore, the book urges the readers (especially language teachers) to explore, in an ecological manner, the factors that influence the effectiveness of their practice. This orientation will ultimately help language teaching practitioners and researchers to build a theoretical framework, through which they can gain a kaleidoscopic picture of online language teaching/learning. Besides, the practices recommended in the book and the relevant literature also encourage teachers to experiment with them and test their effectiveness, which helps teachers to gain a contextualized understanding of online instruction and then take intervention measures to improve their teaching.

Nonetheless, it would be more comprehensive if the book could include some suggestions on such questions as possible tasks that could be used in online courses, classroom interaction, and emotional regulation strategies for both teachers and learners. These suggestions will further ensure the success of conducting online instruction.

Notwithstanding, this is a valuable book that is worth recommending to pre- and in-service language teachers, teacher educators, language teaching researchers, and postgraduates in applied linguistics.

## Author contributions

Both authors read the book simultaneously and then discussed their gains from the book. After reaching the agreement on the merits of the book, LC wrote the first draft of the review, with FW giving constructive suggestions and making some revisions on the subsequent drafts. Therefore, the review is the result of their collective work based on their shared understanding of the book.

## Conflict of interest

The authors declare that the research was conducted in the absence of any commercial or financial relationships that could be construed as a potential conflict of interest.

## Publisher's note

All claims expressed in this article are solely those of the authors and do not necessarily represent those of their affiliated organizations, or those of the publisher, the editors and the reviewers. Any product that may be evaluated in this article, or claim that may be made by its manufacturer, is not guaranteed or endorsed by the publisher.
